# Mutations in the DNA methylation pathway and number of driver mutations predict response to azacitidine in myelodysplastic syndromes

**DOI:** 10.18632/oncotarget.22157

**Published:** 2017-10-27

**Authors:** M. Teresa Cedena, Inmaculada Rapado, Alejandro Santos-Lozano, Rosa Ayala, Esther Onecha, María Abaigar, Esperanza Such, Fernando Ramos, José Cervera, María Díez-Campelo, Guillermo Sanz, Jesús Hernández Rivas, Alejandro Lucía, Joaquin Martínez-López

**Affiliations:** ^1^ Hematology Department, Hospital Universitario 12 Octubre, CNIO, Universidad Complutense, Madrid, Spain; ^2^ Research Institute of Hospital 12 de Octubre (‘i+12’), Madrid, Spain; ^3^ GIDFYS, European University Miguel de Cervantes, Valladolid, Spain; ^4^ IBSAL, Cancer Research Center (USAL-CSIC), Salamanca, Spain; ^5^ Hematology Department, Hospital Universitario La Fe, Valencia, Spain; ^6^ Hematology Department, Hospital Universitario de León, and IBIOMED, Universidad León, León, Spain; ^7^ Genetics Unit, Hospital Universitario La Fe, Valencia, Spain; ^8^ Hematology Department, Hospital Universitario de Salamanca, Salamanca, Spain; ^9^ Universidad Europea de Madrid, Madrid, Spain

**Keywords:** myelodysplastic syndromes, mutational profile, next generation sequencing, hypomethylating agents

## Abstract

We evaluated the association of mutations in 34 candidate genes and response to azacitidine in 84 patients with myelodysplastic syndrome (MDS), with 217 somatic mutations identified by next-generation sequencing. Most patients (93%) had ≥1 mutation (mean=2.6/patient). The overall response rate to azacitidine was 42%. No clinical characteristic was associated with response to azacitidine. However, total number of mutations/patient was negatively associated with overall drug response (odds ratio [OR]: 0.56, 95% confidence interval [CI]: 0.33–0.94; p=0.028), and a positive association was found for having ≥1 mutation in a DNA methylation-related gene: *TET2, DNMT3A, IDH1 and/or IDH2* (OR: 4.76, 95%CI: 1.31–17.27; p=0.017). Mutations in *TP53* (hazard ratio [HR]: 3.88; 95%CI: 1.94–7.75) and *EZH2* (HR: 2.50; 95%CI: 1.23–5.09) were associated with shorter overall survival. Meta-analysis of 6 studies plus present data (n=815 patients) allowed assessment of the association of drug response with mutations in 9 candidate genes: *ASXL1, CBL, EZH2, SF3B1, SRSF2, TET2, DNMT3A, IDH1/2* and *TP53. TET2* mutations predicted a more favorable drug response compared with ‘wild-type’ peers (pooled OR: 1.67, 95%CI: 1.14–2.44; p=0.01). In conclusion, mutations in the DNA methylation pathway, especially *TET2* mutations, and low number of total mutations are associated with a better response to azacitidine.

## INTRODUCTION

Myelodysplastic syndrome (MDS) comprises a heterogeneous group of clinical entities characterized by hematopoietic stem cell damage, leading to peripheral cytopenia and high risk for progression to acute leukemia. At present, the hypomethylating agents azacitidine and decitabine are the only approved drugs for treatment of MDS; however, only 40–50% of patients respond to therapy, although there is individual variability [1]. It is thus important to identify biomarkers that can predict individual treatment response.

Alterations in DNA methylation are involved in the pathogenesis of MDS. Patients show aberrant methylation of cytosine residues in CpG sequences [2, 3] and a DNA hypermethylation profile, particularly in promoter regions of tumor suppressor genes [4, 5]. These genetic alterations can impact patients’ survival [6–10], yet whether they can also influence their response to azacitidine/decitabine treatment has not been clearly elucidated.

DNA methylation status or mutations in genes involved in DNA methylation (e.g., those encoding cyclin dependent kinase inhibitor 2B [*CDKN2B*], estrogen receptor 1 [*ESR1*], immunoglobulin superfamily member 4 [*IGSF4*] or Tet methylcytosine dioxygenase [*TET2*]) are candidates to influence the response to treatment with hypomethylating drugs in MDS [1, 11–13], but there is no unanimity between studies and some data do not support such associations [14–18]. Mutated clone size, mutations in different genes of the same pathway, or interactions between gene mutations, could also affect the response rate to hypomethylating agents and explain, at least in part, heterogeneity between studies as well as individual variability in treatment response.

The main purpose of our study was to evaluate the impact of gene mutations that are candidates to be involved in MDS pathogenesis, detected by next-generation sequencing (NGS), on the response to azacitidine treatment in a cohort of MDS patients. We also performed a meta-analysis, pooling our data with those of available studies, to systematically assess the impact of mutational status on such response.

## RESULTS

### Main clinical characteristics

The demographic and clinical characteristics of the 84 patients are detailed in Table 1. The median follow-up and time from diagnosis to azacitidine treatment was 17 (range 1–93) and 2 months (range 0–22), respectively. Low and intermediate risk patients were treated because of severe cytopenias or excess of blasts. Patients received a median number of 6 cycles of treatment with azacitidine (range 1–55). Fifteen patients with less than 4 cycles of therapy were evaluated as unresponsive due to progression of the disease. At the time of analysis, 29 patients (35%) were continuing on azacitidine therapy. The remaining patients had previously interrupted treatment due to toxicity, progression, or allogeneic stem cell transplantation (one case).

**Table 1:** Clinical characteristics of patients (n=84)

Gender Male/Female	30/54
**Age (years) Median (range)**	69 (49–99)
**Hemoglobin (g/dL)**	
≥10	31 (39%)
8–9.9	33 (41%)
<8	16 (20%)
**Platelets (x10**^**9**^**/L)**	
≥100	29 (36%)
50–99	22 (27%)
<50	30 (37%)
**Leukocytes (x10**^**9**^**/L)**	
≥1.5	76 (95%)
<1.5	4 (5%)
**WHO classification (n (%))**	
RA	1 (1%)
RCMD	18 (22%)
RAEB1	21 (25%)
RAEB2	19 (23%)
5q-	2 (2%)
CMML	7 (8%)
MDS-U	5 (6%)
MDS/AML	11 (13%)
**Cytogenetic risk**	
Very good	2 (2%)
Good	41 (49%)
Intermediate	13 (16%)
Poor	10 (12%)
Very poor	18 (21%)
**IPSS-R category**	
Very low	2 (2%)
Low	15 (18%)
Intermediate	20 (24%)
High	23 (27%)
Very high	21 (25%)
Unclassified	3 (4%)

Abbreviations: AML, acute myeloid leukemia; CMML, chronic myelomonocytic leukemia; IPSS-R, Revised International Prognostic Score System; MDS, myelodysplastic syndrome; RA, refractory anemia; RAEB, refractory anemia with excess blasts; RCMD, refractory cytopenia with multilineage dysplasia; 5q-, MDS with isolated 5q deletion; MDS-U: myelodysplastic syndrome unclassified; WHO, World Health Organization.

### Profile of somatic mutations in MDS patients

We analyzed frequently mutated regions in 34 genes that are candidates to be involved in the pathogenesis of MDS (i.e., with a function in DNA methylation, RNA splicing, histone modulation pathways, or synthesis of transcription/signaling factors).

A total of 217 single nucleotide variants (SNVs) and/or deletions/insertions (indels) were identified by NGS in 78 of 84 patients (93%) (Supplementary Table 1). On average, 2.6 variants (range 0–6) were found per patient. The most frequent mutations (each present, alone or in combination, in ≥10% of patients) were found in the following genes: *TET2* (27%); tumor protein p53 (*TP53*, 20%); DNA methyltransferase 3 alpha (*DNMT3A*, 19%); runt-related transcription factor 1 (*RUNX1*, 18%); enhancer of zeste 2 polycomb repressive complex 2 subunit (*EZH2*, 14%); additional sex combs like 1, transcriptional regulator (*ASXL1*, 13%); U2 small nuclear RNA auxiliary factor 1 (*U2AF1*, 12%); splicing factor 3b subunit 1 (*SF3B1*, 11%); and zinc finger CCCH-type, RNA binding motif and serine/arginine rich 2 (*ZRSR2*, 10%).

The frequencies of these mutations were similar to those published previously [19–21]. Nevertheless, we found a lower frequency of *SF3B1* mutations, associated with good prognosis, and a higher frequency of poor prognosis mutations (*TP53, RUNX1, EZH2, U2AF1, NRAS*). This can be explained by the fact that our series of patients are characterized by therapy requirements, and a high proportion of high-risk patients are included.

### Response to azacitidine and its association with genetic factors

Overall response to azacitidine was 42%, including complete (17%) or partial (5%) remission, and hematologic improvement (20%). In univariate analysis, the overall response rate was significantly and positively associated with the number of azacitidine cycles (odds ratio [OR]=1.17; 95% confidence interval [CI]: 1.11–1.30; p=0.003), but significantly and negatively associated with the total number of mutations (irrespective of the gene/s) per patient (OR=0.64; 95%CI: 0.44–0.94; p=0.022). Both parameters (number of azacitidine cycles and number of mutations) differed between responder and non-responder patients (Table 2).

**Table 2:** Comparison between responder and non-responder patients

	Responders *N*=35	Non-responders *N*=49	*P*-value^*^
**Gender**			
Male/Female	22/13	32/17	*0.817*
**Age (years)**			
Mean± SD	68±8	67±9	*0.589*
**Hemoglobin (g/dl)**			
Mean± SD	9.6±1.4	9.4±2.0	*0.628*
**Platelets (x10**^**9**^**/L)**			
Mean± SD	125.2±140.5	90.9±92.4	*0.189*
**Leukocytes (x10**^**9**^**/L)**			
Mean± SD	6.9±9.1	6.2±8.3	*0.725*
**BM blast (%)**			
Mean± SD	9.6±9.4	9.3±8.9	*0.860*
**WHO classification**			
RCUD, RCMD, CMML, 5q-	10/35 (29%)	18/49 (37%)	*0.434*
RAEB, AML	25/35 (71%)	31/49 (63%)	
**Cytogenetic risk**			
Very good, good, intermediate	24/35 (69%)	32/49 (65%)	*0.754*
Poor, very poor	11/35 (31%)	17/49 (35%)	
**IPSS-R**			
Very low, low, intermediate	17/35 (49%)	20/49 (41%)	*0.480*
High, very high	18/35 (51%)	29/49 (59%)	
**Number of azacitidine cycles**			
Mean± SD	12.91±11.76	6.16±4.03	***<0.001***
**Number of mutations per patient**			
≤2 mutations	27/35 (77%)	27/49 (55%)	***0.038***
>2 mutations	8/35 (23%)	22/49 (45%)	

^*^P- value: Chi square test was used in categorical variables, and Student´s t-test was used for continuous variables. Abbreviations: AML, acute myeloid leukemia; BM, bone marrow; CMML, chronic myelomonocytic leukemia; IPSS-R, Revised International Prognostic Score System; MDS, myelodysplastic syndrome; RA, refractory anemia; RAEB, refractory anemia with excess blasts; RCMD, refractory cytopenia with multilineage dysplasia; 5q-, MDS with isolated 5q deletion; WHO, World Health Organization.

When considering only those genes involved in DNA methylation [*TET2*, *DNMT3A*, isocitrate dehydrogenase 1 (*IDH1*) and 2 (*IDH2)*], or in chromatin modification (*ASXL1*, *EZH2*), we found that both the presence of ≥1 mutation in DNA methylation genes (OR=3.53; 95%CI: 1.10–12.38; p=0.048) and the number of mutations in the DNA methylation pathway group (OR=2.37; 95%CI: 1.10–5.36; p=0.038) were positively and significantly associated with complete response. No such association was found with mutated genes involved in chromatin modification pathways (Table 3).

**Table 3:** Association of gene mutations with overall response to azacitidine

*Univariate analysis*		
Mutated genes	OR (CI 95%)	*P*-value^*^
**DNA methylation factors**		
TET2	1.07 (0.39–2.90)	*0.898*
DNMT3A	1.78 (0.58–5.47)	*0.316*
IDH1	2.91 (0.25–33.41)	*0.391*
IDH2	3.03 (0.52–15.57)	*0.216*
**Histone modulators**		
ASXL1	0.48 (0.12–1.96)	*0.307*
EZH2	0.24 (0.05–1.16)	*0.075*
**Splicing factors**		
SF3B1	0.15 (0.02–1.27)	*0.081*
ZRSR2	0.43 (0.08–2.29)	*0.326*
U2AF1	2.33 (0.60–8.97)	*0.219*
**Transcription factors**		
TP53	0.72 (0.24–2.16)	*0.552*
RUNX1	0.45 (0.13–1.54)	*0.201*
ETV6	0.33 (0.04–3.10)	*0.332*
**Signaling factors**		
JAK2	3.03 (0.52–17.57)	*0.216*
CBL	2.20 (0.35–13.94)	*0.401*
**RAS family**		
KRAS	0.33 (0.04–3.10)	*0.332*
NRAS	0.21 (0.02–1.84)	*0.159*
**Gene interactions**		
TET2mut + ASXL1wt	0.98 (0.33–2.87)	*0.963*
TET2mut +ASXL1mut	1.42 (0.19–10.63)	*0.730*
TET2wt +ASXL1mut	0.21 (0.02–1.84)	*0.159*
DNMT3Amut + ASXL1wt	1.5 (0.47–4.75)	*0.490*
DNMT3Amut + ASXL1mut	2.33	*1.000*
DNMT3Awt + ASXL1mut	0.31 (0.06–1.56)	*0.156*
**At least 1 mutation in DNA methylation genes**	3.53 (1.10–12.38)	0.048^**^
**N° of mutations in DNA methylation genes**	2.37 (1.10–5.36)	0.038^**^

*Multivariate analysis*		
Significant variables	OR (CI 95%)	*P*-value^*^
**N° of cycles of azacitidine**	1.19 (1.10-1.35)	***0.006***
**At least 1 mutation in DNA methylation genes**	4.76 (1.31-17.27)	***0.017***
**N° total of gene mutations**	0.56 (0.33-0.94)	***0.028***

Abbreviations: CI: confidence interval; OR: odds ratio; *mut*: mutated; *wt*: wild-type.

^*^P-value logistic regression results.

^**^ Association with complete response.

In multivariate analysis, the overall response rate remained positively associated with the number of azacitidine cycles (OR=1.19; 95%CI: 1.05–1.35; p=0.006) and with the presence of ≥1 mutation in genes related to DNA methylation pathways (OR=4.76; 95%CI: 1.31–17.27; p=0.017), but negatively so with the total number of mutations per patient (OR=0.56; 95%CI: 0.33–0.94; p=0.028) (Table 3).

Because *TET2* has shown association with azacitidine response in previous studies [1, 12–13], we analyzed the influence of the other genes related to the DNA methylation pathway. When we considered only the cohort of patients with *TET2* wild-type, the multivariate analysis found the same significant variables associated with better response: number of azacitidine cycles (OR: 1.14; 95%CI: 1.01–1.28; p=0.035), number of mutations (OR: 0.44; 95%CI: 0.21–0.89; p=0.023), and mutations in DNA methylation-related genes (considering only *DNMT3A, IDH1 and IDH2*) (OR: 5.80; 95%CI: 1.10–30.85; p=0.039).

**Figure 1:**
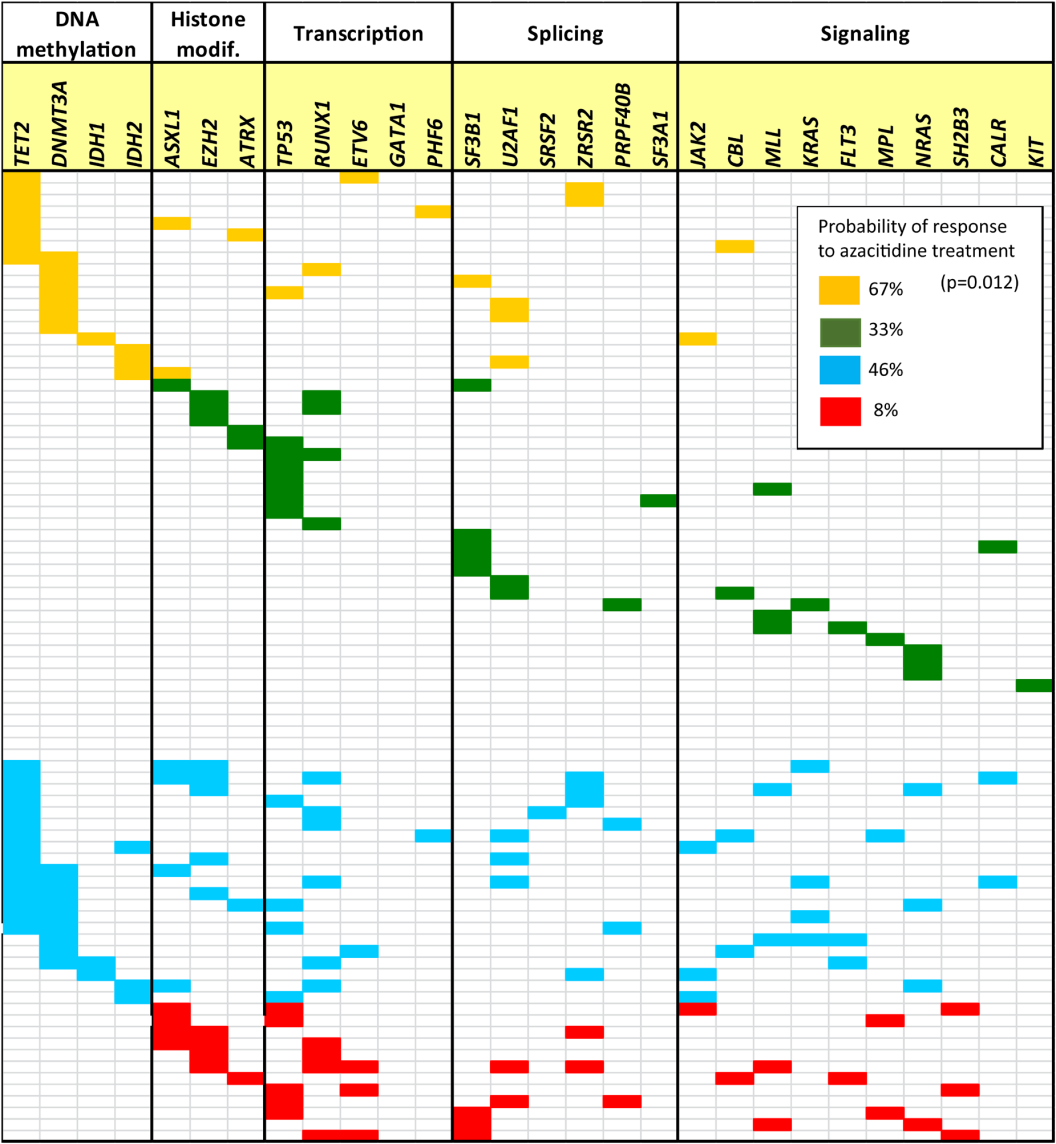
Spectrum of mutations in the 84 patients with myelodysplastic syndrome (MDS) (where each row represents a single patient) Only 28 genes are shown, the remainder of genes analyzed did not present mutations in this cohort of patients (*SF1*, *VHL*, *KDM6A*, *PTEN*, *HRAS*, and *EPOR*). Yellow (group 1): total number of mutations per patient ≤2 and at least ≥1 mutation per patient in DNA methylation pathway; Green (group 2): total number of mutations per patient ≤2, and 0 mutations per patient in DNA methylation genes; Blue (group 3): total number of mutations per patient >2 and at least ≥1 mutation per patient in DNA methylation pathway; and Red (group 4): total number of mutations per patient >2, and 0 mutations per patient in DNA methylation pathway.

The following scoring model was designed using molecular variables at diagnosis: (group 1) total number of mutations per patient ≤2 and at least ≥1 mutation per patient in DNA methylation pathway; (group 2) total number of mutations per patient >2 and at least ≥1 mutation per patient in DNA methylation pathway; (group 3) total number of mutations per patient ≤2 and 0 mutations per patient in DNA methylation genes; and (group 4) total number of mutations per patient >2 and 0 mutations per patient in DNA methylation pathway (Figure 1). The proportion of responders to azacitidine differed across the aforementioned four groups: 67% (12/18) for group 1, 33% (7/21) for group 2, 46% (15/33) for group 3, and 8% (1/12) for group 4 (p=0.012). Significant differences were found between group 1 (higher rate of response than expected, 68%) and group 4 (lower rate of response than expected, 8%) (OR=0.36; 95%CI: 0.17–0.76; p=0.008). The following two variables, mutation number and the presence or absence of mutations in DNA methylation-related genes, contributed to these differences. Indeed, group 1 presented a significant higher probability of response than patients with more than 2 mutations (groups 2 and 4) (OR=0.16; 95%CI: 0.05–0.57; p=0.004), but also than patients with 0 mutations in DNA methylation pathway (groups 3 and 4) (OR=0.28; 95%CI: 0.09–0.88; p=0.029).

### Clinical and genetic predictors of survival

In univariate analysis, considering clinical variables, median of overall survival was significantly higher in the group with platelet count above 100×10^9^/L (p=0.001), low-risk patients according to WHO classification (p=0.014), good cytogenetic risk patients (p=0.016), very low/ low/intermediate IPSS-R (revised international prognostic score system) groups (p=0.007) and responders to azacitidine (p=0.031). Regarding mutations in the 34 studied genes, we found a significant negative association with overall survival for the presence of mutations in either *TP53* (p=0.001) or *RUNX1* (p=0.019)*,* as well as a non-significant trend for the presence of mutations in *EZH2* (p=0.062) (Table 4).

**Table 4:** Prognostic factors for overall survival (univariate survival analysis using the Kaplan-Meier method)

Factors	Overall survival Median *(months)* (95%CI)	*P-* value^*^
**Platelets (x10^9^/L)**		
≥100	34 (16–52)	***0.001***
50-99	23 (13–33)	
<50	16 (7–25)	
**WHO classification**		
RCUD, RCMD, CMML, 5q-	34 (20–48)	***0.014***
RAEB, AML	18 (16–20)	
**Cytogenetic risk**		
Very good, good, intermediate	24 (17–31)	***0.016***
Poor, very poor	17 (12–23)	
**IPSS-R**		
Very low, low, intermediate	29 (21–37)	***0.007***
High, very high	18 (16–20)	
**Response to azacitidine**		
Responders	29 (21–37)	***0.023***
Non-responders	17 (14–20)	
**TP53**		
Unmutated	25 (18–32)	***0.001***
Mutated	11 (7–14)	
**RUNX1**		
Unmutated	24 (17–32)	***0.019***
Mutated	18 (17–19)	
**EZH2**		
Unmutated	23 (21–26)	*0.062*
Mutated	17 (11–23)	
**N° of mutations per patient**		
≤2	26 (14–37)	***0.002***
>2	17 (12–22)	

^*^for Kaplan–Meier (log rank) test results.

We further analyzed whether the presence of >2 mutations (i.e., above the mean number of mutations per patient in our cohort, 2.6) influenced survival, finding that, irrespective of the gene in question, having >2 mutations was associated with a shorter survival as compared with having ≤2 mutations (p=0.002) (Table 4).

**Figure 2:**
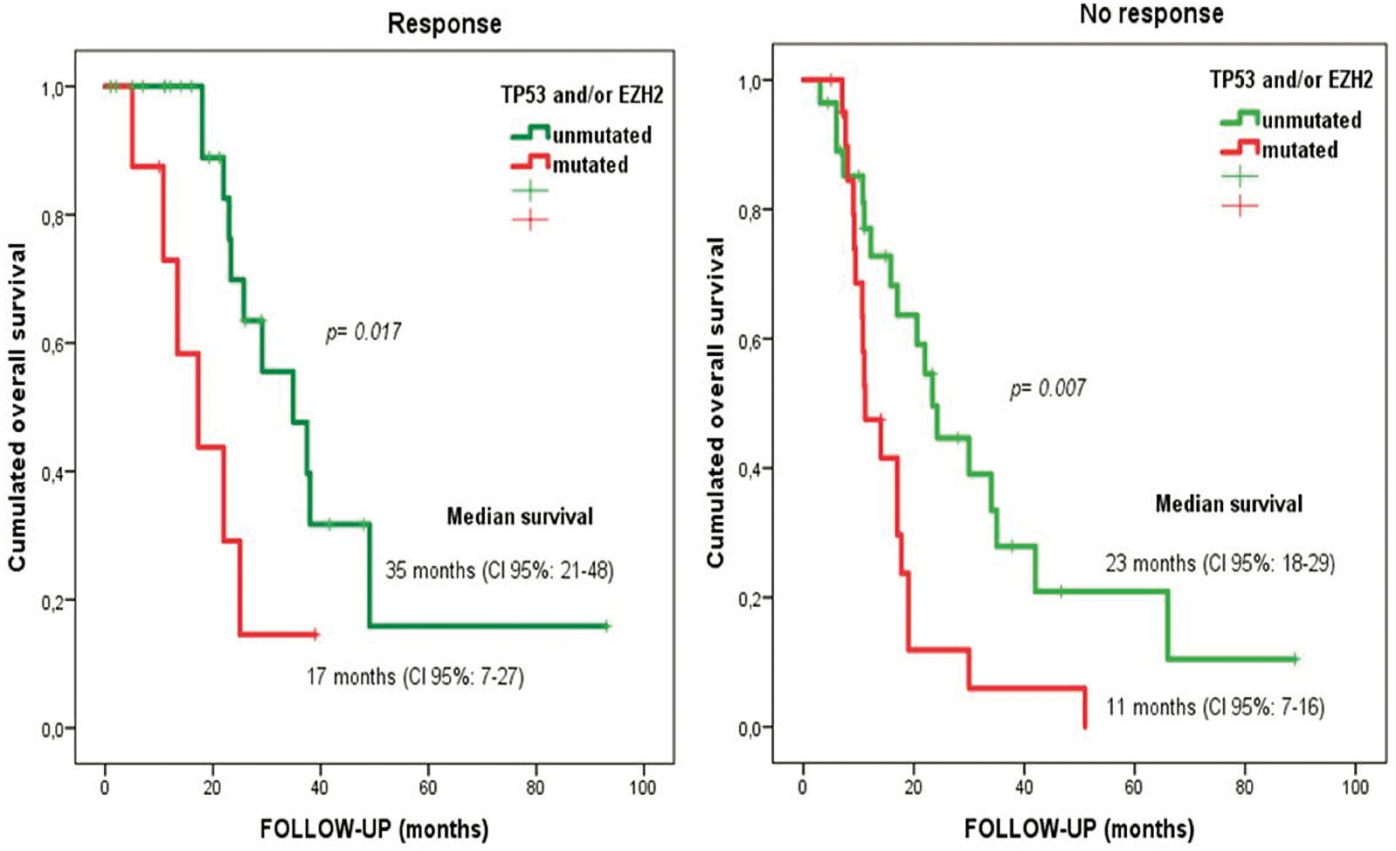
Overall survival for responder and non-responder patients depending on *EZH2* and/or *TP53* mutational status Median overall survival of 35 months versus 17 months (in *TP53* and *EZH2* wild-type versus *TP53* and/or *EZH2* mutated patients, respectively) in responders to azacitidine (log rank statistic p=0.017). Median overall survival of 23 months versus 11 months (in *TP53* and *EZH2* wild-type versus mutated patients) in non-responders to azacitidine (log rank statistic p=0.007).

In multivariate analysis, having ≥1 mutation in *TP53* [hazard ratio (HR)=3.88; 95CI%: 1.94–7.75; *p*<0.001] or *EZH2* remained the most important prognostic factor for overall survival (HR=2.50; 95%CI: 1.23–5.09; p=0.012). Interestingly, the impact of mutational status of *TP53* and *EZH2* in overall survival was independent of the response to azacitidine (Figure 2).

### Meta-analysis results

**Figure 3:**
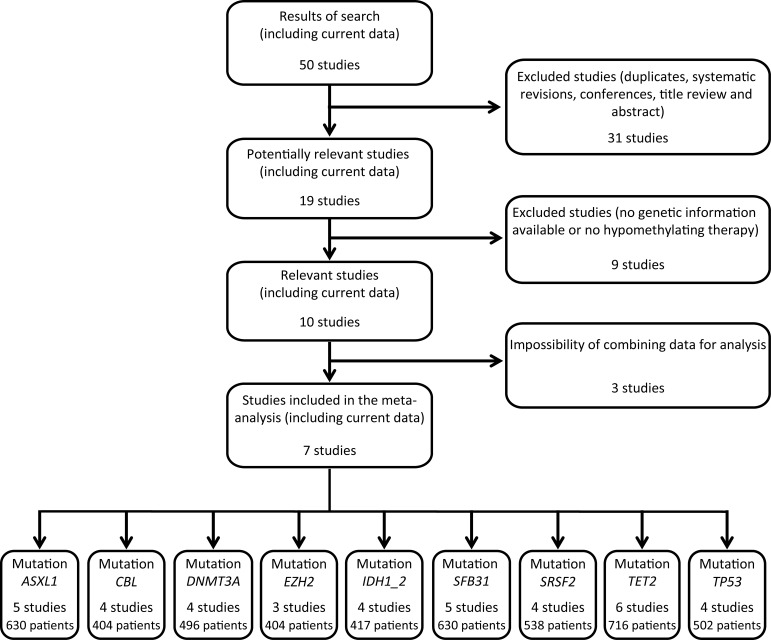
Flow chart of studies included and excluded in the meta-analysis Fifty papers were identified in the electronic databases *PubMed, Embase, Cochrane library* and *Web of Science.* Revisions and studies with no genetic information or no hypomethylating therapy were excluded. Finally, seven studies (including current data) containing mutational status of 9 genes were included in the meta-analysis.

The results from 6 previous studies [1, 12, 13, 17, 22, 23] and the present data, involving a total of 815 patients with mutation analysis in at least one of the 9 candidate genes studied, were pooled in the meta-analysis, allowing us to assess the association of drug response with mutations in 9 genes [*ASXL1,* Cbl proto-oncogene (*CBL*), *DNMT3A*, *EZH2*, *IDH1/IDH2*, *SF3B1*, serine and arginine rich splicing factor 2 (*SRSF2*)*, TET2* and *TP53*] (Figure 3). All 7 studies were of high quality according to the Newcastle-Ottawa scale.

**Figure 4:**
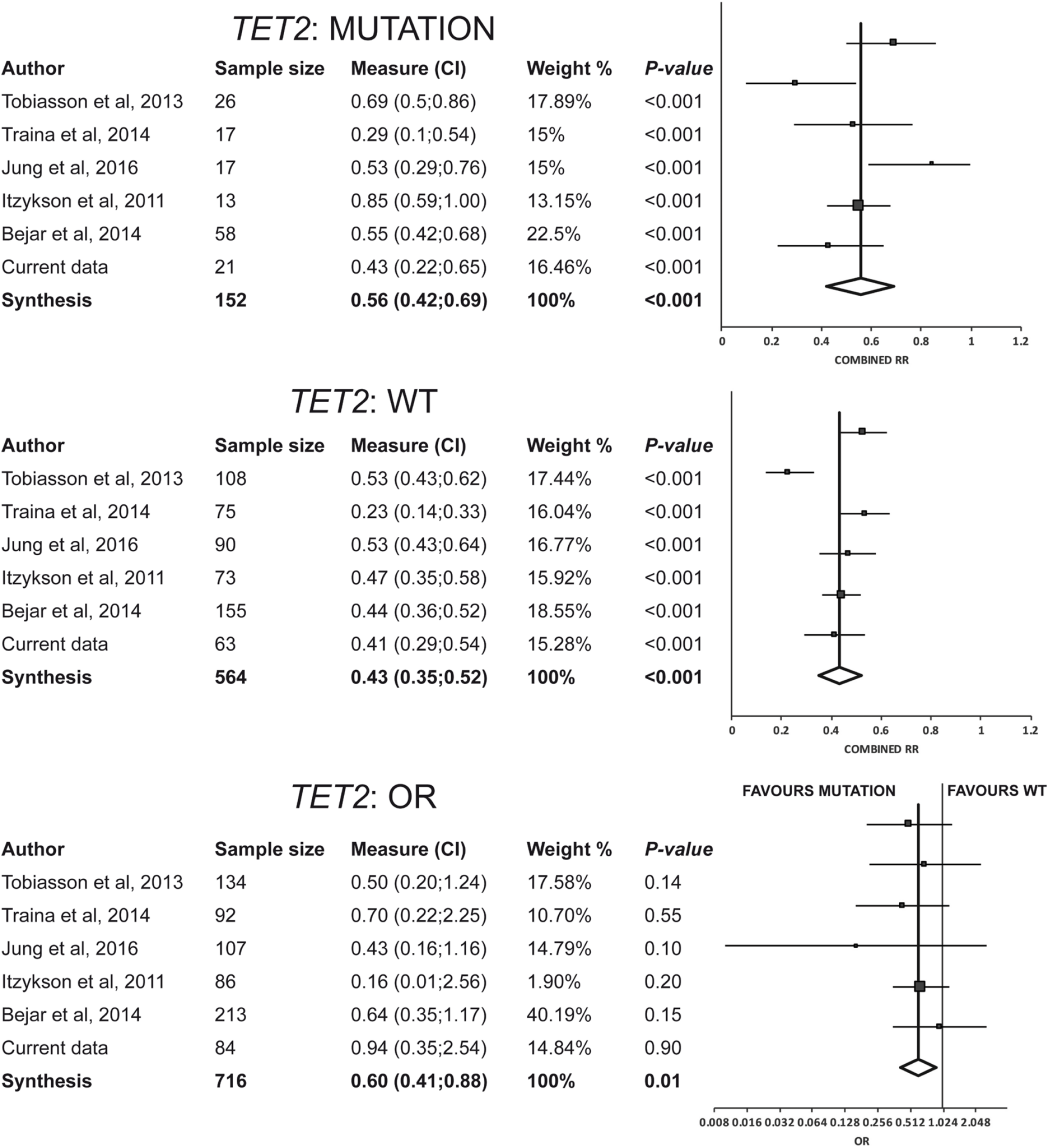
Results of meta-analysis for *TET2* Combined response rate for patients with *TET2* mutated (response rate: 56%; 95%CI: 42–69%) resulted significantly higher than that of the remaining patients with *TET2* wild-type (RR: 43%; 95%CI: 35–52%). The pooled odds ratio of response was favourable to *TET2* mutations (pooled OR=1.67, 95%CI: 1.14–2.44, *p*=0.01). Abbreviations: OR, odds ratio: RR, response rate; WT, wild-type.

The combined response rate of patients (22% of total) with ≥1 *TET2* mutation was 56% (95%CI: 42%–69%), with significant heterogeneity among studies (I^2^=60.3%, Q=12.6) and no evidence of publication bias (p=0.937) (Figure 4). Such combined response was significantly higher (p<0.01) than that of the remaining patients harboring no *TET2* mutation (43%) (95%CI: 35%–52%), with significant heterogeneity among studies (I^2^=77.4%, Q=22.1) and no evidence of publication bias (p=0.4) (Figure 4). Combining the data on *TET2* from the aforementioned studies with ours (total n=716 participants with available data of *TET2* mutation status), the combined response rate to treatment was more favorable in those patients with ≥1 genetic mutation in *TET2* than in those with no *TET2* mutation (pooled OR=1.67, 95%CI: 1.14–2.44, p=0.01) (Figure 4). There was no evidence of publication bias (p=0.94) or heterogeneity among the studies (I^2^=0.00%, Q=3.99).

No other significant association was found between gene mutations and treatment response (all p>0.1) (Supplementary File 2).

## DISCUSSION

We have found that the profile of several gene mutations identified at diagnosis may represent a useful predictive biomarker of the response to azacitidine therapy in patients with MDS. Accordingly, patients with a total of ≤2 somatic mutations in candidate genes and ≥1 mutation in genes of the DNA methylation pathway (*TET2, DNMT3A, IDH1, and IDH2*) had an overall response rate of 67%, which is well above the typical expected value (40–50%). By contrast, patients with >2 somatic mutations in total but having none in the aforementioned pathway represented the group with the lowest probability of response to treatment with azacitidine (8%). Meta-analysis revealed that the *TET2* gene is the strongest biomarker of clinical response.

High-sensitivity sequencing of a specific panel of myeloid-related genes has allowed us to identify somatic mutations in >90% of the MDS patients we studied. The inclusion of molecular markers in prognostic scores for MDS, together with other clinical and genetic data, is likely to lead to a better prediction of patient outcomes in the near future. Previous studies have assessed the role of candidate gene mutations in the context of MDS therapy, including response to erythropoietic stimulating agents [24], to hypomethylating agents [1, 12, 13, 17, 22, 23, 25, 26], or in the bone marrow transplantation setting [27–29]. In the present study, no clinical characteristic was associated with overall response to azacitidine, and the same was true for any single mutated gene. Yet, our results support the notion that the DNA methylation pathway is influential in the response to therapy in MDS. Some previous studies have found an association of *TET2* mutations with treatment response to azacitidine when the histone modifier *ASXL1* was not mutated or when a mutated clone over 10% was considered [13], but other authors found no such association [17, 22]. When we considered all genes related to the DNA methylation pathway (*TET2*, *DNMT3A*, *IDH1*, and *IDH2*), the presence of mutation/s in any of them was related to overall treatment response. No additional prognostic values were found when we combined the aforementioned genes with other epigenetic genes, such as *ASXL1*.

A particularly interesting finding of our clinical study was that the total number of mutations in candidate genes per patient was inversely related to the response to azacitidine, with previous research showing a close relationship between survival, but not of response to this drug [20]. Although we have not performed a comprehensive mutational analysis (only 34 genes were studied), all genes with mutation frequencies above 2% in myelodysplastic syndromes according to the European LeukemiaNet recommendations were included [30]. Moreover, the association we found between higher total number of mutations per patient and lower response rate to azacitidine remained after adjusting for IPSS-R. The molecular complexity of the disease both at diagnosis and during its progression can determine the probability of response to therapy. Further, we hypothesize that acquisition of additional mutations could be one of the reasons for loss of treatment response, which should be explored in future research. Those patients with a total of >2 somatic mutations but none in the DNA methylation pathway had the lowest probability to respond to azacitidine (i.e., of only 8%). Thus, alternative therapies should be considered in this group of poor responders due to the low probability of successful therapy with this drug.

Searching for prognostic factors of survival, we based our analysis on clinical and molecular data. In this regard, we found two genes, *TP53* and *EZH2*, with a negative impact on overall survival, with this effect remaining after adjusting for IPSS-R risk. Both genes have been previously studied in high-risk MDS [19–21], but no association was reported with the response rate to therapy. Other authors have also described a negative influence of *TP53* mutations in the survival patients with MDS or acute myeloid leukemia (AML), despite no effect on the response rate to azacitidine therapy [26, 31, 32]. A study by Welch and colleagues [23] found higher response rates among patients with *TP53* mutations than those among patients with wild-type *TP53*; although 77% of the patients were diagnosed with AML and not MDS, and the treatment regimen, 10-day course of decitabine, is unusual for MDS. Thus, whereas previously available and present data support treatment with hypomethylating agents in patients with *TP53* and/or *EZH2* mutations, their expected low survival rate advocates the need to explore other treatment strategies in this group of patients.

An additional strength and novelty of our study is the systematic review of the literature and subsequent meta-analysis, allowing us to confirm the significant role of *TET2* mutations in response to azacitidine. Despite the heterogeneity among studies, the high number of patients in each group (mutated and ‘wild-type’ patients by each gene analyzed) supports the validity and generalizability of our meta-analysis.

In conclusion, a low total number of mutations in candidate genes coupled with one or more mutations in DNA methylation-related genes predict a better response to azacitidine. Furthermore, meta-analysis identified the *TET2* gene as the strongest biomarker of treatment success. Mutational profiling of candidate genes provides important prognostic information for MDS patients under therapy. The presence of mutations in the DNA methylation pathway and the number of driver mutations are predictors of response to hypomethylating agents. Larger collaborative studies are needed to confirm and extend our findings.

## MATERIALS AND METHODS

### Cohort study

#### Patient samples

A total of 84 patients with MDS from three Spanish institutions who had been (or were still) receiving azacitidine were included in the analysis. Bone marrow samples were obtained from patients at diagnosis and processed following standard work-up protocols. All patients gave their written informed consent to biobank samples. The following clinical characteristics were considered: gender, age, World Health Organization (WHO) classification, cytogenetic risk, IPSS-R, response to azacitidine (according to International Working group response criteria) [33] and outcome. Duration of follow-up was censored at the time of transplantation, and in the remaining patients until loss or death.

#### High sensitivity targeted sequencing and mutation analysis

DNA was extracted from bone marrow mononuclear cells. High-depth NGS was performed using an Ion Torrent Proton™ sequencer (Life Technologies, Palo Alto, CA). In each procedure performed, the total number of reads was ∼2 million, with an average depth of coverage over 2, 000 reads by amplicon, along with high uniformity for all fragments (91.6%). Data analyses were performed using Ion Reporter 4.4 software (Life Technologies), which identified SNVs and indels. We used Ion Reporter default parameters and filtered out variants with a total coverage <40 reads and an allelic coverage <10 reads. All variants reported had a variant allele frequency above 4%. Variants with a minor allelic frequency >0.01 in the general population according to the Single Nucleotide Polymorphism database and/or 5000 Exome Sequencing Project were rejected as possible polymorphisms [34]. Variants were categorized according to cancer mutation databases and algorithms for computational prediction of functional impact of variants (see Supplementary Information and Supplementary Table 1 ): type 1; variants of known clinical significance, type 2: variants of potential clinical significance, and type 3: variants of unknown clinical significance.

#### Statistical analysis

Clinical characteristics of the patients were compared with the χ^2^ test (or Fisher´s test if ≥80% of the cells of the cross table had an expected frequency <5) for categorical variables, and Student’s t test for continuous variables (or its non-parametric equivalent if data did not follow a normal distribution). The association between clinical variables, including mutational status, and the response to azacitidine, was investigated using logistic regression. Cox proportional hazard models and Kaplan-Meier curves were used to assess association of variables (clinical data and mutations) with patient overall survival. Statistical significance level was set at 0.05 and analyses were conducted with SPSS 21.0 software (SPSS Inc., Chicago, IL).

### Pooled meta-analysis

#### Eligibility criteria, information sources and search strategy

Relevant papers were identified in the electronic databases *PubMed, Embase, Cochrane library* and *Web of Science,* using the following keywords (chosen according to the scientific literature): “*myelodysplastic syndrome/s”,* “*mutation*”*, “hypomethylating”,* as well as any possible combinations of these terms, up to February 20, 2017. The title and abstracts of the selected articles were reviewed to determine the potential eligibility for meta-analysis.

The criteria for including a study in the systematic review were the following: patients with diagnosis of MDS who were treated with one hypomethylating agent (either azacitidine or decitabine), assessment of mutational analysis with sequencing platforms and information on the response rate in ‘mutated’ *versus* ‘wild-type’ patients for genes that are candidates to be involved in MDS.

#### Data extraction and quality assessment

We followed the Preferred Reporting Items for Systematic Reviews and Meta-Analyses (PRISMA) guidelines [35] (Supplementary File 1). We collected the following items from each study, if available: number of patients with gene mutation/s, number of patients with a ‘wild-type’ genotype (i.e., 0 mutations), and response rate of these two types of patients. Two independent reviewers extracted the data. The Newcastle-Ottawa scale was used to assess the quality of each study included in the meta-analysis.

### Statistical analysis

A random effects model meta-analysis of proportions was used to estimate the ‘combined response rate’ (95% CI to the treatment of MDS with hypomethylating therapy). Group rates were transformed using the Freeman-Tukey double arc-sine method with a standard error and sample size. The pooled results were then back-transformed to give estimates and forest plots in the original scale and these were reported. The pooled OR (95%CI) of response to treatment if having ≥1 gene mutation as compared with having the wild-type genotype was estimated using a weighted random-effect model.

Egger’s regression test was employed to identify the presence of publication bias, and heterogeneity among studies was assessed using the Cochrane Q-test and the I^2^ index. The level of significance was set at 0.05 and statistical analyses were performed using MIX Pro software version 2.0 [36].

The data discussed in this publication have been deposited in the NCBI Gene Expression Omnibus [37] and are accessible through GEO Series accession number GSE54920

(http://www.ncbi.nlm.nih.gov/geo/query/acc.cgi?acc=GSE54920) and GEO SubSeries accession numbers: SRP102906.

Supplementary information is available at the *Oncotarget* web site.

## SUPPLEMENTARY MATERIALS TABLES









## Abbreviations

MDS: myelodysplastic syndrome
SNV: single nucleotide variant
NGS: next-generation sequencing
OR: odds ratio
CI: confidence interval
IPSS-R: revised international prognostic score system
AML: acute myeloid leukemia
WHO: World Health Organization
