# Correction: Mutations in the DNA methylation pathway and number of driver mutations predict response to azacitidine in myelodysplastic syndromes

**DOI:** 10.18632/oncotarget.25856

**Published:** 2018-07-20

**Authors:** M. Teresa Cedena, Inmaculada Rapado, Alejandro Santos-Lozano, Rosa Ayala, Esther Onecha, María Abaigar, Esperanza Such, Fernando Ramos, José Cervera, María Díez-Campelo, Guillermo Sanz, Jesús Hernández Rivas, Alejandro Lucía, Joaquin Martínez-López

**Affiliations:** ^1^ Hematology Department, Hospital Universitario 12 Octubre, CNIO, Universidad Complutense, Madrid, Spain; ^2^ Research Institute of Hospital 12 de Octubre (‘i+12’), Madrid, Spain; ^3^ GIDFYS, European University Miguel de Cervantes, Valladolid, Spain; ^4^ IBSAL, Cancer Research Center (USAL-CSIC), Salamanca, Spain; ^5^ Hematology Department, Hospital Universitario La Fe, Valencia, Spain; ^6^ Hematology Department, Hospital Universitario de León, and IBIOMED, Universidad León, León, Spain; ^7^ Genetics Unit, Hospital Universitario La Fe, Valencia, Spain; ^8^ Hematology Department, Hospital Universitario de Salamanca, Salamanca, Spain; ^9^ Universidad Europea de Madrid, Madrid, Spain

**This article has been corrected:** In the Materials and Methods section, the data access link is incorrect. In addition, reference 37 was also listed incorrectly. The proper information is given below:

Materials and Methods: The data discussed in this publication have been deposited in the NCBI Sequence Read Archive (SRA) [37] and are accessible through (http://www.ncbi.nlm.nih.gov/sra/SRP102906).

References: 37. Leinonen R, Sugawara H, Shumway M. The sequence read archive. Nucleic Acids Res. 2011; 39 (Database issue): D19-D21.

Original article: Oncotarget. 2017; 8:106948-106961. https://doi.org/10.18632/oncotarget.22157

