# Supplementary material for: Mutations in the DNA methylation pathway and number of driver mutations predict response to azacitidine in myelodysplastic syndromes
Source: Oncotarget. 2017 Oct 27;8(63):106948–61. doi: 10.18632/oncotarget.22157 (PMC5739787; doi:10.18632/oncotarget.22157)
Supplement: Supplementary file 1 [file oncotarget-08-106948-s001.pdf]

Mutations in the DNA methylation pathway and number of driver mutations predict response to azacitidine in myelodysplastic syndromes

Supplementary Table 1: Descriptions of variants analysed and categorized according to cancer mutation databases and algorithms for computational prediction of functional impact of variants

See Supplementary File 1

Supplementary File 1: PRISMA Checklist

See Supplementary File 2

Supplementary File 2: Supplemental Information

See Supplementary File 3
