# Supplementary material for: Mutations in the DNA methylation pathway and number of driver mutations predict response to azacitidine in myelodysplastic syndromes
Source: Oncotarget. 2017 Oct 27;8(63):106948–61. doi: 10.18632/oncotarget.22157 (PMC5739787; doi:10.18632/oncotarget.22157)
Supplement: Supplementary file 4 [file oncotarget-08-106948-s004.docx]

**VARIANT CATEGORIZATION**

According to cancer mutation databases and algorithms for computational prediction of functional impact of variants ^(1,2,3,4)^, we established the following variant categorization:

Type I: variants of known clinical significance

- Variants described as disease-related in Clinvar,
- Inserts and deletions in frameshit, and nonsense mutations,
- Variants described in Cosmic, and pathogenic meaning in all prediction algorithms (SIFT, Polyphen-2, FATHMM).

Type II: variants of potent clinical significance

- Variants described as pathogenic in at least two of the prediction algorithms.

Type III: variants of unknown clinical significance

- Not enough information available to determine pathogenicity.

^1^ Landrum MJ, Lee JM, Benson M, Brown G, Chao C, Chitipiralla S, et al. [ClinVar: public archive of interpretations of clinically relevant variants.](https://www.ncbi.nlm.nih.gov/pubmed/26582918) Nucleic Acids Res. 2016 Jan 4;44(D1):D862-8.

[www.ncbi.nlm.nih.gov/clinvar](http://www.ncbi.nlm.nih.gov/clinvar)

^2^ Forbes SA, Beare D, Gunasekaran P, Leung K, Bindal N, Boutselakis H et al. [COSMIC: exploring the world's knowledge of somatic mutations in human cancer.](https://www.ncbi.nlm.nih.gov/pubmed/25355519) Nucleic Acids Res. 2015 Jan;43(Database issue):D805-11.

COSMIC v80, released 13-FEB-17. cancer.sanger.ac.uk

^3^ Vaser R, Adusumalli S, Leng SN, Sikic M, Ng PC. [SIFT missense predictions for genomes.](https://www.ncbi.nlm.nih.gov/pubmed/26633127) Nat Protoc. 2016 Jan;11(1):1-9.

SIFT Human DB (release 63)

^4^ Adzhubei IA, Schmidt S, Peshkin L, Ramensky VE, Gerasimova A, Bork P, et al. [A method and server for predicting damaging missense mutations.](https://www.ncbi.nlm.nih.gov/pubmed/20354512) Nat Methods. 2010 Apr;7(4):248-9.

Polyphen-2 .

**SEQUENCED CHROMOSOME REGIONS IN THE GENES OF THE STUDY**

| **Gene** | **Sequenced Region** |
| --- | --- |
| ***MPL*** | Exons and splicing regions sequenced |
| ***NRAS*** | Exons and splicing regions sequenced |
| ***DNMT3A*** | Exons and splicing regions sequenced |
| ***SF3B1*** | Exons and splicing regions sequenced |
| ***IDH1*** | Exons and splicing regions sequenced |
| ***VHL*** | Exons and splicing regions sequenced |
| ***KIT*** | Exons and splicing regions sequenced |
| ***TET2*** | Exons and splicing regions sequenced |
| ***EZH2*** | Exons and splicing regions sequenced |
| ***JAK2*** | Exons and splicing regions sequenced |
| ***PTEN*** | Exons and splicing regions sequenced |
| ***HRAS*** | Exons and splicing regions sequenced |
| ***SF1*** | Exons and splicing regions sequenced |
| ***MLL*** | Exons and splicing regions sequenced |
| ***CBL*** | Exons and splicing regions sequenced |
| ***ETV6*** | Exons and splicing regions sequenced |
| ***KRAS*** | Exons and splicing regions sequenced |
| ***PRPF40B*** | Exons and splicing regions sequenced |
| ***SH2B3*** | Exons and splicing regions sequenced |
| ***FLT3*** | Exons and splicing regions sequenced |
| ***IDH2*** | Exons and splicing regions sequenced |
| ***TP53*** | Exons and splicing regions sequenced |
| ***SRSF2*** | Exons and splicing regions sequenced |
| ***EPOR*** | Exons and splicing regions sequenced |
| ***CALR*** | Exons and splicing regions sequenced |
| ***ASXL1*** | Exons and splicing regions sequenced |
| ***RUNX1*** | Exons and splicing regions sequenced |
| ***U2AF1*** | Exons and splicing regions sequenced |
| ***SF3A1*** | Exons and splicing regions sequenced |
| ***ZRSR2*** | Exons and splicing regions sequenced |
| ***KDM6A*** | Exons and splicing regions sequenced |
| ***PHF6*** | Exons and splicing regions sequenced |
| ***GATA1*** | Exons and splicing regions sequenced |
| ***ATRX*** | Exons and splicing regions sequenced |

**META-ANALYSIS RESULTS**

Seven studies involving 815 patients and current data were included in the meta-analysis. Of a maximum 9-point score in the New Castle Ottawa scale, all studies met high quality score.

***TET2* mutation**

Six studies (1, 12–13, 17, 23, present data) have evaluated the response to treatment of patients with/without the *TET2* mutation, which has been found in a total of 152 patients. The results of the meta-analysis show a significant combined response rate of 56% in patients with the mutation (95%CI: 42, 69; p<0.001), with significant heterogeneity among studies (I^2^=60.3%, Q=12.6) and no evidence of publication bias (p=0.937). The combined response rate in 564 patients without the *TET2* mutation was 43% (95%CI: 35, 52), with significant heterogeneity among studies (I^2^=77.4%, Q=22.1) but no evidence of publication bias (p=0.45).

Combining the data from the aforementioned six studies (total n=716 participants), the pooled OR of response to treatment was 0.60 (95%CI: 0.41, 0.88, p=0.01) favorable to genetic mutation. There was no evidence of publication bias (p=0.75) or heterogeneity among the studies (I^2^=0.0%, Q=2.4).

***DNMT3A* mutation**

Four studies (1, 13, 23, present data) have evaluated the response to treatment of patients with/without the *DNMT3A* mutation, which has been found in a total of 66 patients. The results of the meta-analysis show a significant combined response rate of 47% in patients with the *DNMT3A* mutation (95%CI: 34, 60; p<0.001), with significant heterogeneity among studies (I^2^=87.7%, Q=24.5) but no evidence of publication bias (p=0.734). The combined response rate in 430 patients without the *DNMT3A* mutation was 40% (95%CI: 27, 54), with significant heterogeneity among studies (I^2^=87.6%, Q=24.1) but no evidence of publication bias (p=0.127).

The pooled OR (total n=496 participants) of response to treatment was 0.81 (95%CI: 0.42, 1.54; p=0.51) favorable to genetic mutation. Although there was no evidence of publication bias (p=0.51), there was heterogeneity among studies (I^2^=25.8%, Q=4.04).

***IDH1/2* mutation**

Four studies (1, 17, 23, present data) have evaluated the response to treatment of patients with/without the *IDH1/2* mutation, which has been found in a total of 37 patients. The results of the meta-analysis show a significant combined response rate of 57% in patients with the mutation (95%CI: 39, 74; p<0.001), with significant homogeneity among studies (I^2^=0%, Q=1.3) and no evidence of publication bias (p=0.587). The combined response rate in 380 patients without mutation was 42% (95%CI: 27, 58), with significant heterogeneity among studies (I^2^=89.6%, Q=28.9) but no evidence of publication bias (p=0.120).

The pooled OR (total n=417 participants) of responding to treatment was 0.62 (95%CI: 0.30, 1.25, p=0.18) favorable to genetic mutation. There was no evidence of publication bias (p=0.311) or heterogeneity among the studies (I^2^=0.0%, Q=2.72).

***ASXL1* mutation**

Of the seven studies included in the meta-analysis, five (1, 13, 17, 23, present data) have assessed the response to treatment of patients with/without the *ASXL1* mutation. The *ASXL1* mutation has been found in a total of 183 patients. The results of the meta-analysis show a significant combined response rate of 44% in patients with the mutation (95%CI: 32, 57; p<0.001), with significant heterogeneity among studies (I^2^=53.7%, Q=8.6) but no evidence of publication bias (p=0.81). The combined response rate in 447 patients without mutation was 45% (95%CI: 34, 56), with significant heterogeneity among studies (I^2^=82.3%, Q=22.6) but no evidence of publication bias (p=0.15).

When combining the data from the studies (total n=630 participants), the pooled OR of response to treatment was 1.06 (95%CI: 0.73, 1.53; *p*=0.77) favorable to ‘wild-type’ patients. There was no evidence of publication bias (p=0.79) or heterogeneity among studies (I^2^=0.0%, Q=3.8).

***EZH2* mutation**

Three studies (13, 23, present data) have assessed the response to treatment of patients with/without the *EZH2* mutation, which has been found in 43 patients in total. The results of the meta-analysis show a significant combined response rate of 41% in patients with the mutation (95%CI: 18, 65; p<0.001), with homogeneity among studies (I^2^=0.0%, Q=1.0) and no evidence of publication bias (p=0.11). The combined response rate in 361 patients without mutation was 48% (95%CI: 43, 53), with significant heterogeneity among studies (I^2^=0.1%, Q=1.3) and no evidence of publication bias (p=0.15.

Combining the data from the studies (total n=404 participants), the pooled OR of response to treatment was 1.25 (95%CI: 0.50, 3.12; p=0.63) favorable to ‘wild-type’ patients. There was no evidence of publication bias (p=0.51), but there was heterogeneity among the studies (I^2^=37.4%, Q=3.2).

***TP53* mutation**

Four studies (13, 23–24, present data) have evaluated the response to treatment of patients with/without the *TP53* mutation, which has been found in a total of 90 patients. The results of the meta-analysis showed a significant combined response rate of 71% in patients with the *TP53* mutation (95%CI: 34, 97; p<0.001), with significant heterogeneity among studies (I^2^=91.0%, Q=33.3) and no evidence of publication bias (p=0.616). The combined response rate in 413 patients without the *DNMT3A* mutation was 48% (95%CI: 43, 53), with significant heterogeneity among studies (I^2^=50.1%, Q=13.1) and no evidence of publication bias (p=0.37).

The pooled OR (total n=502 participants) of response to treatment was 0.51 (95%CI: 0.18, 1.47, p=0.21) favorable to genetic mutation. Although there was no evidence of publication bias (p=0.36), there was heterogeneity among studies (I^2^=64.76%, Q=8.51).

***SF3B1* mutation**

Five studies (1, 13, 17, 23, present data) have assessed the response to treatment of patients with/without the *SF3B1* mutation, which has been found in a total of 73 patients. The results of the meta-analysis show a significant combined response rate of 37% in patients with the mutation (95%CI: 22, 53; p<0.001), with significant heterogeneity among studies (I^2^=83.9%, Q=24.8) but no evidence of publication bias (p=0.05). The combined response rate in 557 patients without mutation was 45% (95%CI: 35, 56), with significant heterogeneity among studies (I^2^=87.9%, Q=24.8) but no evidence of publication bias (p=0.05).

When combining the data from the studies (total n=630 participants), the pooled OR of response to treatment was 1.27 (95%CI: 0.75, 2.16; *p*=0.37) favorable to ‘wild-type’ patients. Although there was no evidence of publication bias (p=0.771), there was heterogeneity among the studies (I^2^=0.0%, Q=3.9).

***SRSF2* mutation**

Four studies (13, 17, 23, present data) have evaluated the response to treatment of patients with/without the *SRSF2* mutation, which has been found in 61 patients in total. The results of the meta-analysis show a significant combined response rate of 58% in patients with the *SRSF2* mutation (CI: 41, 75; p<0.001), with significant homogeneity among studies (I^2^=0.0%, Q=1.4) and no evidence of publication bias (p=0.17). The combined response rate in 477 patients without the *SRSF2* mutation was 48% (95%CI: 42, 54), with significant heterogeneity among studies (I^2^=42.6%, Q=5.2) but no evidence of publication bias (p=0.73).

The pooled OR (total n=538 participants) of responding to treatment was 0.83 (95%CI: 0.48, 1.44, p=0.51) favorable to genetic mutation. There was no evidence of publication bias (p=0.66) or heterogeneity among the studies (I^2^=0.0%, Q=1.3).

***CBL* mutation**

Four studies (1, 13, 23, present data) have assessed the response to treatment of patients with/without the *CBL* mutation, which has been found in 25 patients in total. The combined response rate was 30% (95%CI: 0.06, 0.59), with homogeneity among studies (I^2^=41.8%, Q=3.4) and no evidence of publication bias (p=0.30). The combined response rate in 469 patients without mutation was 40% (95%CI: 3, 51), with significant heterogeneity among studies (I^2^=87.7%, Q=16.4) but no evidence of publication bias (p=0.06).

The pooled OR (total n=404 participants) of responding to treatment was 1.64 (95%CI: 0.48, 5.58; *p*=0.43) favorable to ‘wild-type’ patients. There was no evidence of publication bias (p=0.24) but there was heterogeneity among the studies (I^2^=42.18%, Q=3.46).
